# Correction: Infectious reactivation of cytomegalovirus explaining age- and sex-specific patterns of seroprevalence

**DOI:** 10.1371/journal.pcbi.1007146

**Published:** 2019-06-12

**Authors:** The PLOS Computational Biology Staff

## Notice of republication

This article was republished on January 17, 2018, to correct errors that were introduced during the typesetting process. The publisher apologizes for the errors. Please download this article again to view the correct version.

## Supporting information

S1 File Republished, corrected article. (PDF)
