# Supplementary material for: Correction: Infectious reactivation of cytomegalovirus explaining age- and sex-specific patterns of seroprevalence
Source: PLoS Comput Biol. 2019 Jun 12;15(6):e1007146. doi: 10.1371/journal.pcbi.1007146 (PMC6561530; doi:10.1371/journal.pcbi.1007146)
Supplement: S1 File — (PDF) [file pcbi.1007146.s001.pdf]

PLOS | COMPUTATIONAL BIOLOGY

RESEARCH ARTICLE

Infectious reactivation of cytomegalovirus explaining age- and sex-specific patterns of seroprevalence

Michiel van Boven[1]*, Jan van de Kassteele[1], Marjolein J. Korndewal[1,2], Christiaan H. van Dorp[1,3], Mirjam Kretzschmar[1,4], Fiona van der Klis[1], Hester E. de Melker[1], Ann C. Vossen[2], Debbie van Baarle[1]

1 Centre for Infectious Disease Control, National Institute for Public Health and the Environment, Bilthoven, the Netherlands, **2** Leiden University Medical Center, Department of Medical Microbiology, Leiden, the Netherlands, **3** Theoretical Biology and Bioinformatics, Utrecht University, Utrecht, the Netherlands, **4** Julius Center for Health Sciences and Primary Care, University Medical Center Utrecht, Utrecht, the Netherlands

* michiel.van.boven@rivm.nl

Abstract

Human cytomegalovirus (CMV) is a herpes virus with poorly understood transmission dynamics. Person-to-person transmission is thought to occur primarily through transfer of saliva or urine, but no quantitative estimates are available for the contribution of different infection routes. Using data from a large population-based serological study (n = 5,179), we provide quantitative estimates of key epidemiological parameters, including the transmissibility of primary infection, reactivation, and re-infection. Mixture models are fitted to age- and sex-specific antibody response data from the Netherlands, showing that the data can be described by a model with three distributions of antibody measurements, i.e. uninfected, infected, and infected with increased antibody concentration. Estimates of seroprevalence increase gradually with age, such that at 80 years 73% (95%CrI: 64%-78%) of females and 62% (95%CrI: 55%-68%) of males are infected, while 57% (95%CrI: 47%-67%) of females and 37% (95%CrI: 28%-46%) of males have increased antibody concentration. Merging the statistical analyses with transmission models, we find that models with infectious reactivation (i.e. reactivation that can lead to the virus being transmitted to a novel host) fit the data significantly better than models without infectious reactivation. Estimated reactivation rates increase from low values in children to 2%-4% per year in women older than 50 years. The results advance a hypothesis in which transmission from adults after infectious reactivation is a key driver of transmission. We discuss the implications for control strategies aimed at reducing CMV infection in vulnerable groups.

Author summary

Human cytomegalovirus (CMV) is a herpes virus causing lifelong infection. In high-income countries, the probability of infection increases gradually with age such that at old age up to 100% of the population is infected. CMV is thought to be transmitted mainly by

Check for updates

OPEN ACCESS

Citation: van Boven M, van de Kassteele J, Korndewal MJ, van Dorp CH, Kretzschmar M, van der Klis F, et al. (2017) Infectious reactivation of cytomegalovirus explaining age- and sex-specific patterns of seroprevalence. PLoS Comput Biol 13(9): e1005719. https://doi.org/10.1371/journal.pcbi.1005719

Editor: Samuel Alizon, CNRS, FRANCE

Received: February 3, 2017

Accepted: August 2, 2017

Published: September 26, 2017

Copyright: © 2017 van Boven et al. This is an open access article distributed under the terms of the Creative Commons Attribution License, which permits unrestricted use, distribution, and reproduction in any medium, provided the original author and source are credited.

Data Availability Statement: Data are available at GitHub (github.com/mvboven/cmv-serology).

Funding: This work was supported by the Dutch Ministry of Health, Welfare and Sport and the Netherlands Organisation for Scientific Research (grants 645.000.002 and 823.02.014). The funders had no role in study design, data collection and analysis, decision to publish, or preparation of the manuscript.

Competing interests: The authors have declared that no competing interests exist.

transfer of saliva or urine, but little quantitative evidence is available about the transmission dynamics. We analyze serological data to estimate age- and sex-specific rates of infection, re-infection, and reactivation. The analyses show that infectious reactivation (i.e. reactivation of the virus in an infected person that is sufficient for it to be transmitted to another person) is essential to explain the data. We propose that infectious reactivation in adults is an important driver of transmission of CMV.

Introduction

Human cytomegalovirus (CMV) is a highly prevalent herpesvirus that infects between 30% and 100% of persons in populations throughout the world [1]. Usually thought to be a relatively benign persistent infection, CMV is able to cause serious disease in the immunocompromised and offspring of pregnant women with an active infection [2–5]. CMV also has been implicated in a variety of diseases in healthy persons [4, 6–8], and plays a role in aging of the immune system [9–12], perhaps thereby reducing the effectiveness of vaccination in older persons [13–15].

Although the importance of CMV to public health is acknowledged, and even though the development and registration of a vaccine has been declared a priority [16, 17], little quantitative information is available on the transmission dynamics of CMV. At present, the only population-level data derive from serological studies, aiming to uncover which part of the population is infected at what age. These studies show that i) a sizable fraction of infants is infected perinatally (before 6 months of age), ii) seroprevalence increases gradually with age and is usually higher in females than in males, and iii) the probability of seropositivity is associated with both ethnicity and socioeconomic status, with non-western ethnicity and lower socioeconomic status being associated with higher rates of seropositivity [1, 18–21].

CMV infection has a profound impact on the human immune system. Most prominently, it is able to mould the T cell immune repertoire, in particular by expansion of the CMV-specific CD8+ memory T cell pool, a phenomenon called memory inflation [12]. Similar result have been found for memory B cell immunity [22]. With regard to humoral immune responses, high levels of CMV-specific IgG antibodies are increasingly considered a biomarker for lack of control by the immune system of the host, and have been associated with high probability of reactivation ([23, 24], see [12] and references therein). In view of this, it is not surprising that evidence is accumulating of an association between high levels of CMV-specific IgG antibodies, inflammation, vascular disease, and mortality [6, 7].

Person-to-person transmission of CMV from an infected to an uninfected person can occur from a primary infected person, or from a person who is experiencing a reactivation episode or from a person who has been reinfected [4]. Here, we analyze data from a large-scale serological study to obtain quantitative estimates of the relative importance of these transmission routes [21]. We fit mixture models linked to age- and sex-specific transmission models to the data to study the ability of different hypotheses explaining the serological data. Specifically, we quantify the incidence and transmissibility of primary infection, re-infection, and reactivation. Throughout, our premise is that measurements of antibody concentrations provide information on whether or not a person has been infected, and whether or not re-infection or reactivation have occurred. Persons with low measurements are considered uninfected (susceptible), while persons with intermediate and high antibody concentrations are infected with and without subsequent re-infection or reactivation, respectively.

The analyses show that infectious reactivation in adults is necessary to explain the data, and is expected to be an important driver of transmission. The results have implications for control of CMV by vaccination, but also in the broader context of T cell immune memory inflation, vascular disease, and immunosenescence [12, 25, 26].

Methods

Ethics statement

The study was approved by the Medical Ethics Testing Committee of the foundation of therapeutic evaluation of medicines (METC-STEG) in Almere, the Netherlands (clinical trial number: ISRCTN 20164309). All participants or their legal representatives had given written informed consent.

Study design

The analyses make use of sera from a cross-sectional population-based study carried out in the Netherlands in 2006-2007. Details have been published elsewhere [21, 27]. Briefly, 40 municipalities distributed over five geographic regions of the Netherlands were randomly selected with probabilities proportional to their population size, and an age-stratified sample was drawn from the population register. A total of 19,781 persons were invited to complete a questionnaire and donate a blood sample. Serum samples and questionnaires were obtained from 6,382 participants. To exclude the interference of maternal antibodies, we restrict analyses to sera from persons older than 6 months (6,215 samples). We further select Dutch persons and migrants of Western ethnicity to preclude confounding by ethnicity (5,179 samples) and stratify the data by sex [21], yielding 2,842 and 2,337 samples from female and male participants, respectively. The data are available at github.com/mvboven/cmv-serology.

Antibody assay

We use the ETI-CYTOK-G PLUS (DiaSorin, Saluggia, Italy) Elisa to detect CMV-specific IgG antibodies. The assay yields continuous measurements (henceforth called 'antibody concentration'). A small number of samples is right-censored (140 persons). We perform a Box-Cox transformation of the data ($\lambda = 0.3$), yielding a distribution of low antibody concentrations ($-2.8 < x \leq -0.5$) that is approximately normal. According to the provider of the assay, samples with (transformed) measurement lower than -0.8 U/ml should be considered uninfected, while samples with measurement greater or equal than -0.8 U/ml should be classified as infected. Right-censoring is applied to the 140 samples above the upper limit of 3.41 U/ml. The data with model fit (see below) are shown in Fig 1.

Mixture model

The data are analyzed statistically using a mixture model with sex- and age-specific mixing functions. We distinguish three distributions, describing samples of low (susceptible, S), intermediate (latently infected, L), and high (latently infected with increased antibodies, B) antibody concentrations. The L and B distributions are modeled using normal distributions with means and standard deviations independent of age and sex. The S distribution is modeled by a mixture of a spike and a normal distribution (an inflated normal distribution), as there appears a spike at -2.91 U/ml in the data (263 persons). In this way, samples with concentration at the spike belong to the susceptible component with probability 1.

We model the probability of each of the three outcomes in terms of log-odds, taking the probability of being in the S component as reference. This allows us to write the log-odds of

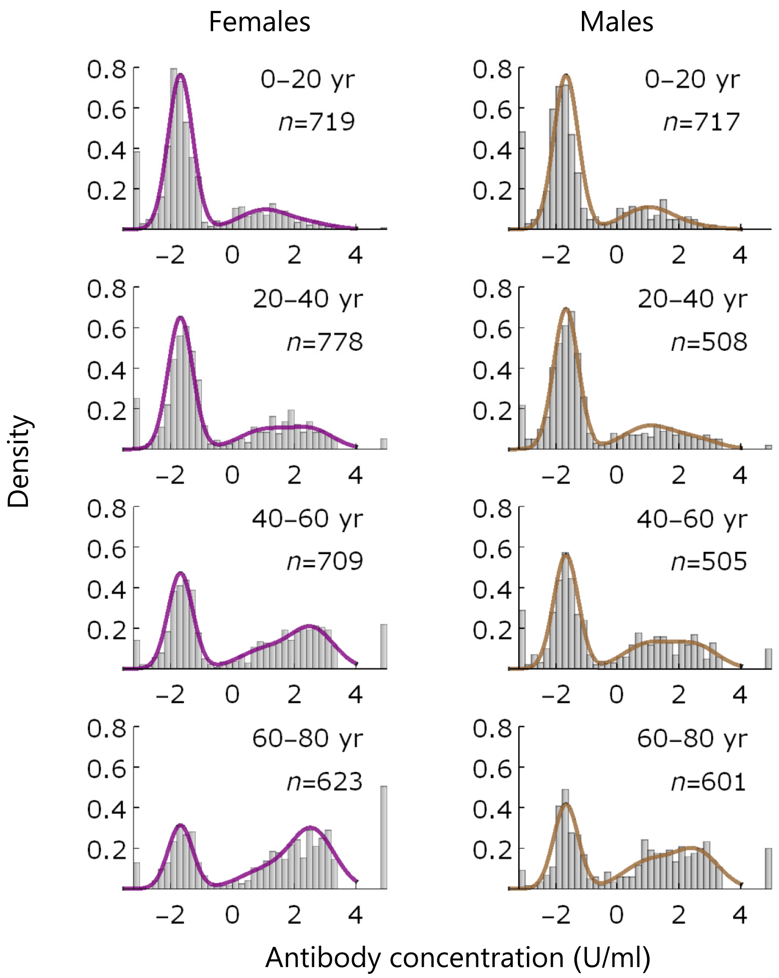

Fig 1. Data and model fit. Data (histograms) and model fit (lines) of IgG antibody measurements by age group and sex. Left- and right-hand panels show results for females (purple) and males (brown), respectively. The leftmost bars at -2.9 contain samples that are assumed uninfected, and the rightmost bars at 4.5 contain samples that are right-censored (with concentration >3.41 U/ml; Methods). Insets show the age group and number of samples.

https://doi.org/10.1371/journal.pcbi.1005719.g001

being in component L or B as linear functions of age and sex. The design matrix of the resulting multinomial logistic model consists of natural cubic splines with interior knots at 20, 40 and 60 years and boundary knots at 0 and 80 years. Hence, the mixing functions (prevalences) have flexible shape, which allows these to be optimally informed by the data. In the results, sex is put in the model as main effect, as analyses show no improvement in fit when including age by sex interaction.

We estimate parameters in a Bayesian framework using R and JAGS [28, 29]. Non-informative normal prior distributions are set on the means of the three component distributions ($\mathcal{N}(0, 0.001)$) (mean and precision). Label switching is prevented by prior ordering of the means. The precisions of the components are given flat Gamma prior distributions (Γ(0.5, 0.005)). The spline parameters are also given non-informative normal prior distributions ($\mathcal{N}(0, 0.001)$). We apply a QR-decomposition to the design matrix to improve mixing and run 10 MCMC chains in parallel, yielding a total of 10,000 samples. We apply an 1/10 thinning to give a well-mixed 1,000 samples from the posterior distribution.

Transmission model and scenarios

Next to the mixture model analyses, we estimate parameters of transmission models to investigate the ability of different transmission hypotheses explaining the data. To facilitate comparison between transmission models, take the medians of the estimated mixture distributions as input. In line with the above, we focus on a sex- and age-structured model in which persons are probabilistically classified as uninfected (S), latently infected (L), and latently infected after reactivation or re-infection (B). As the infectious period is short relative to the lifespan of the host (weeks versus decades), the infectious periods are modeled implicitly using the short-disease approximation [30]. Further, we focus on the endemic equilibrium of the transmission model so that all variables are time-independent [30, 31]. Fig 2 shows a schematic of the model. For sexes $i \in \{♀, ♂\}$, the differential equations for the age-specific relative frequencies $S(a)$, $L(a)$, and $B(a)$ ($S(a) + L(a) + B(a) = 1$) are given by

$$\begin{aligned} \frac{dS^i(a)}{da} &= -\lambda^i(a)S^i(a) \\ \frac{dL^i(a)}{da} &= \lambda^i(a)S^i(a) - (\rho^i(a) + z\,\lambda^i(a))L^i(a) \\ \frac{dB^i(a)}{da} &= (\rho^i(a) + z\,\lambda^i(a))L^i(a)\ , \end{aligned} \tag{1}$$

with forces of infection

$$\lambda^i(a) = \sum_{j\in\{♀,♂\}} \int_0^M c^{ij}(a,a')(\beta_1\lambda^j(a')S^j(a') + \beta_2(\rho^j(a') + z\lambda^j(a'))L^j(a'))da'\,. \tag{2}$$

In Eqs (1) and (2), $z\lambda^j(a)$ and $\rho^j(a)$ are the age-specific re-infection and reactivation rates, z is the susceptibility to re-infection of latently infected persons relative to the susceptibility of uninfected persons ($0 \leq z \leq 1$), $c^{ij}(a, a')$ represents the contact rate between persons of age a' and sex j, and those of age a and sex i [32, 33], β_1 and β_2 are proportionality parameters determining the transmissibility of primary infection and reactivation/re-infection, and M is the maximum age. As the data do not extend beyond 80 years we take $M = 80$ years. Notice that $\lambda^j(a)S^j(a)$ and $(\rho^j(a) + z\,\lambda^j(a))L^j(a)$ are the incidence of primary infection and the incidence of reactivation and re-infection, so that $\beta_1\lambda^j(a)S^j(a)$ and $\beta_2(\rho^j(a) + z\,\lambda^j(a))L^j(a)$ are the infectious output generated by primary infection and reactivation/re-infection, respectively [30].

As in earlier studies, contact rates are hard-wired into the model using data on social contact patterns, thereby adopting the social contact hypothesis [32–34]. Here we use the mixing matrix based on reported physical contacts [32]. The discretized contact function and demographic data are available at github.com/mvboven/cmv-serology.

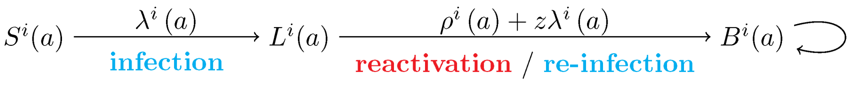

Fig 2. Schematic of the transmission model. $S^i(a)$ denotes the proportion of uninfected persons of age a and sex i ($i \in \{♀, ♂\}$), and $L^i(a)$ and $B^i(a)$ are the corresponding proportions of infected persons without and with increased antibodies, respectively. The infection and re-infection rates are given by $\lambda^i(a)$ and $z\lambda^i(a)$, and the reactivation rates are given by $\rho^i(a)$. We consider model scenarios with and without reactivation/re-infection in the B compartment (i.e. including or excluding the loop to the right of B).

https://doi.org/10.1371/journal.pcbi.1005719.g002

Below, we consider a suite of simplifications and variations of the full model specified by Eqs (1) and (2). In the simplifications, we assume that (i) there is no re-infection ($z = 0$), (ii) there is no reactivation ($\rho^i(0) = 0$), or (iii) reactivation and re-infection are not infectious ($\beta_2 = 0$). We also consider a variation of the model in which re-infection and reactivation do not only occur upon transition from L to B, but also in the B compartment. In these models the infectious output generated by reactivation and re-infection in Eq (2) ($\beta_2(\rho^j(a') + z\lambda^j(a'))L^j(a')$) is replaced by $\beta_2(\rho^j(a') + z\lambda^j(a'))(L^j(a') + B^j(a'))$.

Solution and discretization

The differential equations can be solved in terms of the forces of infection using the variation of constants method. Here we assume, based on results of the mixture model, that a non-negligible fraction of infants is infected in the first six months of life and the fraction infected is equal in female and male infants [21]. Hence, we have $S^{♀}(0) = S^{♂}(0) = S_0$, $L^{♂}(0) = L^{♀}(0) = 1 - S_0$, and $B^{♀}(0) = B^{♂}(0) = 0$ as initial conditions, and the solution of (1) is given by

$$\begin{aligned} S^i(a) &= S_0^i e^{-\int_0^a \lambda(\tau)d\tau} \\ L^i(a) &= (1 - S_0^i)e^{-\int_0^a \rho(\tau)+z\lambda(\tau)d\tau} + S_0^i \int_0^a \lambda(\tau)e^{-\int_0^\tau \lambda(\tau')d\tau' - \int_\tau^a \rho(\tau')+z\lambda(\tau')d\tau'} d\tau\,. \end{aligned} \tag{3}$$

Insertion of Eq (3) in Eq (2) yields two integral equations for the age-specific forces of infection in females and males [34–37]. These equations cannot be solved explicitly in general. It is possible, however, to solve the equations for specific functions.

Here, we assume that reactivation and contact rates are constant on certain predefined age-intervals. From Eq (2), it then follows that the force of infection is piecewise constant as well. Throughout, we consider age intervals of fixed size $\Delta a = 5$ years, so that the limits of the $n = M/\Delta a = 16$ age classes are defined by the vector $\mathbf{a} = (0, \Delta a, 2\Delta a, \ldots, n\Delta a)$. Hence, the j-th class ($j = 1, \ldots, n$) contains all persons with age in the interval $[a_{[j]}, a_{[j+1]})$, where $a_{[j]}$ denotes the j-th element of $\mathbf{a}$. Subsequently, the forces of infection $\lambda^i(a)$ and reactivation rates $\rho^i(a)$ are replaced by their counterparts λ_j^i and ρ_j^i. Similarly, $S^i(a)$, $L^i(a)$, and $B^i(a)$ at the borders of the age-intervals are given by S_j^i, L_j^i, and B_j^i. Insertion in Eq (3) and integrating over the (constant) rates yields

$$\begin{aligned} S_j^i &= S_0\ e^{-\Delta a \sum_{k=1}^j \lambda_k^i} \\ L_j^i &= (1 - S_0)\ e^{-\Delta a \sum_{k=1}^j \rho_k^i + z\lambda_k^i} + \\ & \quad S_0 \sum_{k=1}^j \lambda_k^i \frac{e^{-\Delta a(\rho_k^i + z\lambda_k^i)} - e^{-\Delta a \lambda_k^i}}{(1-z)\lambda_k^i - \rho_k^i} e^{-\Delta a(\sum_{\ell=1}^{k-1} \lambda_\ell^i - \sum_{\ell=k+1}^j \rho_\ell^i + z\lambda_\ell^i)}\,, \end{aligned} \tag{4}$$

where $i \in \{♀, ♂\}$ and $B_j^i = 1 - S_j^i - L_j^i$. Insertion of Eq (4) in Eq (2) and making use of the fact that the cumulative incidences of infection and reactivation/re-infection in age class j are given by $\int_{a_{[j]}}^{a_{[j+1]}} \lambda^i(a)S^i(a)da = S^i(a_{[j]}) - S^i(a_{[j+1]})$ and $B^i(a_{[j+1]}) - B^i(a_{[j]})$, yields 32 equations (16 per sex) for the 32 forces of infection.

Estimation and model selection

As in the mixture model with spline mixing parameters, the log-likelihood of each observation is given by a mixture distribution, where the spline functions are replaced by $S^i(a)$, $L^i(a)$, and $B^i(a)$. For instance, the likelihood contribution of a sample with antibody measurement c in a

person of sex i and age a is given by

$$S^i(a)f_S(c) + L^i(a)f_L(c) + B^i(a)f_B(c) \ ,$$

where $S^i(a)$, $L^i(a)$, and $B^i(a)$ are the age specific prevalences in sex i, and $f_S(c)$, $f_L(c)$, and $f_B(c)$ are the densities of the mixture distributions at antibody concentration c.

In both sexes, reactivation rates are modeled by piecewise constant functions with steps at 20 and 50 years, i.e. with rates that are constant on the intervals [0, 20), [20, 50), and [50, 80) years. Hence, the reactivation rates are characterized by three parameters in each sex, viz. $\rho^i_{[0,20)}$, $\rho^i_{[20,50)}$, and $\rho^i_{[50,80)}$ ($i \in \{♀, ♂\}$).

Bayesian parameter estimates are obtained using Markov chain Monte Carlo (MCMC). Initially, results were obtained using tailored Mathematica code, using a single-component random walk metropolis algorithm while solving the consistency equations for the forces of infection using a Quasi-Newton (secant) method. As this became exceedingly slow for specific models, we recoded the models using Hamiltonian Monte Carlo with Stan (mc-stan.org). Here, the discretized equations for the forces of infection (2) are solved by specifying that the differences between the left- and right-hand sides are small, and approximately $\mathcal{N}(0, 10^{-4})$ (mean and scale) distributed. Cross-checking of the two methods yielded very similar results. All programs are available at github.com/mvboven/cmv-serology.

Prior distributions of the parameters are as follows: $\beta_1 \sim \mathcal{N}(0.1, 10)$ (mean and scale), $\beta_2 \sim \mathcal{N}(0.1, 10)$, $z \sim \mathcal{U}(0, 1)$, $\mu_\rho \sim \mathcal{N}(0, 10)$, $1/\sigma_\rho \sim \mathcal{N}(0, 10)$, and $\rho^i_x \sim \mathcal{N}(\mu_\rho, \sigma_\rho)$ for all i and x. Whenever applicable, distributions are truncated to be positive. With these prior parameter distributions, the joint posterior distribution is strongly dominated by the data. Ten chains of 3,000 iterations are run in parallel, of which the first 500 iterations (warmup) are discarded. We apply 1/5 thinning, yielding a total of 5,000 samples per model scenario. For all parameters, effective sample sizes usually lie between 3,000 and 4,500. Convergence of chains is assessed visually, and by assessment of the empirical variance within and between chains [38]. To prevent the occurrence of divergent transitions we set ADAPT_DELTA = 0.99. Parameter estimates and bounds of credible intervals are represented by 2.5, 50, and 97.5 percentiles of the posterior samples. Results are usually obtained in 1-3 hours on a personal computer.

Model selection is based on WAIC, a measure for predictive performance, and WBIC, a measure for identifying the most likely model generating the data [39–41]. WAIC is obtained directly from the posterior likelihood using the R-package LOO (cran.r-project.org). WBIC is calculated in a separate run as the average log likelihood over the posterior samples, using a sampling 'temperature' determined by the number of observations [39].

Results

Classification

Fig 1 presents the data stratified by sex and age, with fit of the statistical model. The data and model fit show peaks at low antibody measurements (-2.9 U/ml and ≈-2 U/ml), corresponding to uninfected persons (denoted by S). In both sexes, there is a third peak at higher measurements (1-3 U/ml) that shifts to higher values with increasing age. This peak is composed of persons who are infected (denoted by L) and persons who are infected with high antibody concentrations (denoted by B). Overall, the model appears to describe the data well.

This is confirmed in Fig 3, which shows the estimated components of the mixture distribution and diagnostic characteristics of the classification. The component distribution of uninfected persons hardly overlaps with the two component distributions for infected persons,

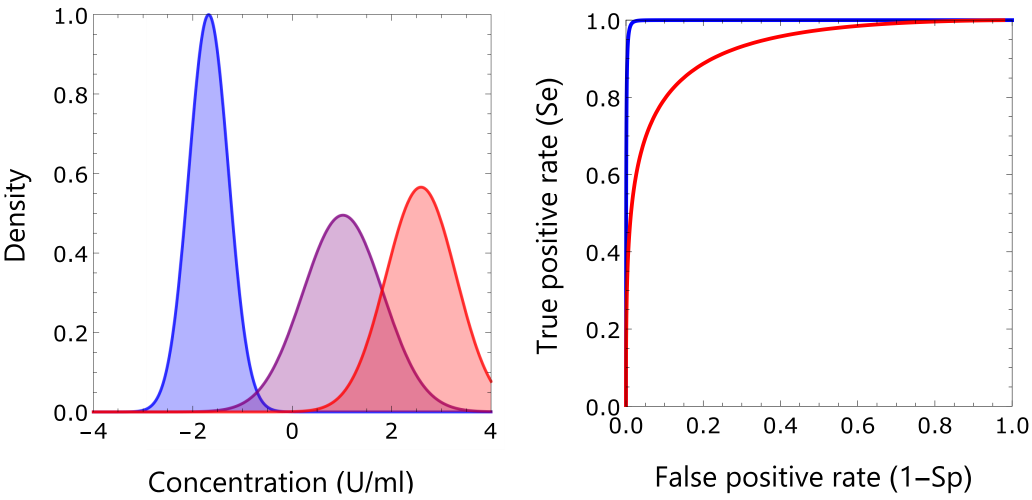

Fig 3. Classification of samples. Shown are the estimated components of the mixture distribution using the parameter posterior medians (left-hand panel; blue: susceptible; purple: infected; red: infected with increased antibody concentration), and receiver operating characteristic of binary classifications taking these estimates as ground truth (right-hand panel). The maximal Youden index for classification of uninfected versus infected persons is 0.97 at antibody concentration -0.70 U/ml, with sensitivity 0.99 and specificity 0.98. This value corresponds well with the threshold for infection of -0.8 U/ml provided by the supplier of the assay. The maximal Youden index for classification of persons with increased antibody concentration is 0.71 at antibody concentration 1.81 U/ml, with sensitivity 0.84 and specificity 0.87.

https://doi.org/10.1371/journal.pcbi.1005719.g003

while there is some overlap between the distributions of infected persons. This can be made more precise using detection theory. Specifically, in Fig 3 we graph the specificity *Sp* (the probability of correctly classifying a negative subject) and sensitivity *Se* (the probability of correctly classifying a positive subject) in a receiver operating characteristic (ROC) graph with antibody concentration specifying a cut-off for binary classification as parameter [42–44]. Subsequently, we use the maximal Youden index (i.e. $\max(Se + Sp - 1)$) to choose an optimal cut-off, and find that classification of persons as uninfected versus infected is near perfect (Youden index: 0.97, at cut-off -0.70 U/ml), while classification of persons with high antibody concentrations is good (Youden index: 0.71, at cut-off 1.81 U/ml). These results show that the classification is supported by the data (i.e. has high probability yielding an informed decision).

We further investigate whether mixture models with fewer or more components are able to provide an even better description of the data, and found that a model with two mixture components does not perform well (ΔWAIC = 300.2 in favor of the three-component mixture distribution), while performance of models with four components depends sensitively on choice of prior distribution of the fourth distribution, and often yields broad posterior antibody distributions with small estimated prevalence that overlap with the other three component distributions. Hence, a mixture model with three components gives an optimal description of the data.

Prevalence estimation

Fig 4 shows the estimated prevalences in females and males as a function of age [42–44]. The prevalence of uninfected persons decreases gradually with age, from approximately 0.80 in infants (females: 0.81, 95%CrI: 0.77-0.85; males: 0.80, 95%CrI: 0.76-0.84) to 0.27 (95%CrI: 0.22-0.34) and 0.38 (95%CrI: 0.32-0.45) at 80 years in females and males, respectively. In both females and males the latently infected prevalence remains approximately constant, ranging from 0.15 to 0.20 in females and from 0.18 to 0.28 in males. In contrast, the prevalence of persons with increased antibodies increases strongly with age, especially in females. In fact, the

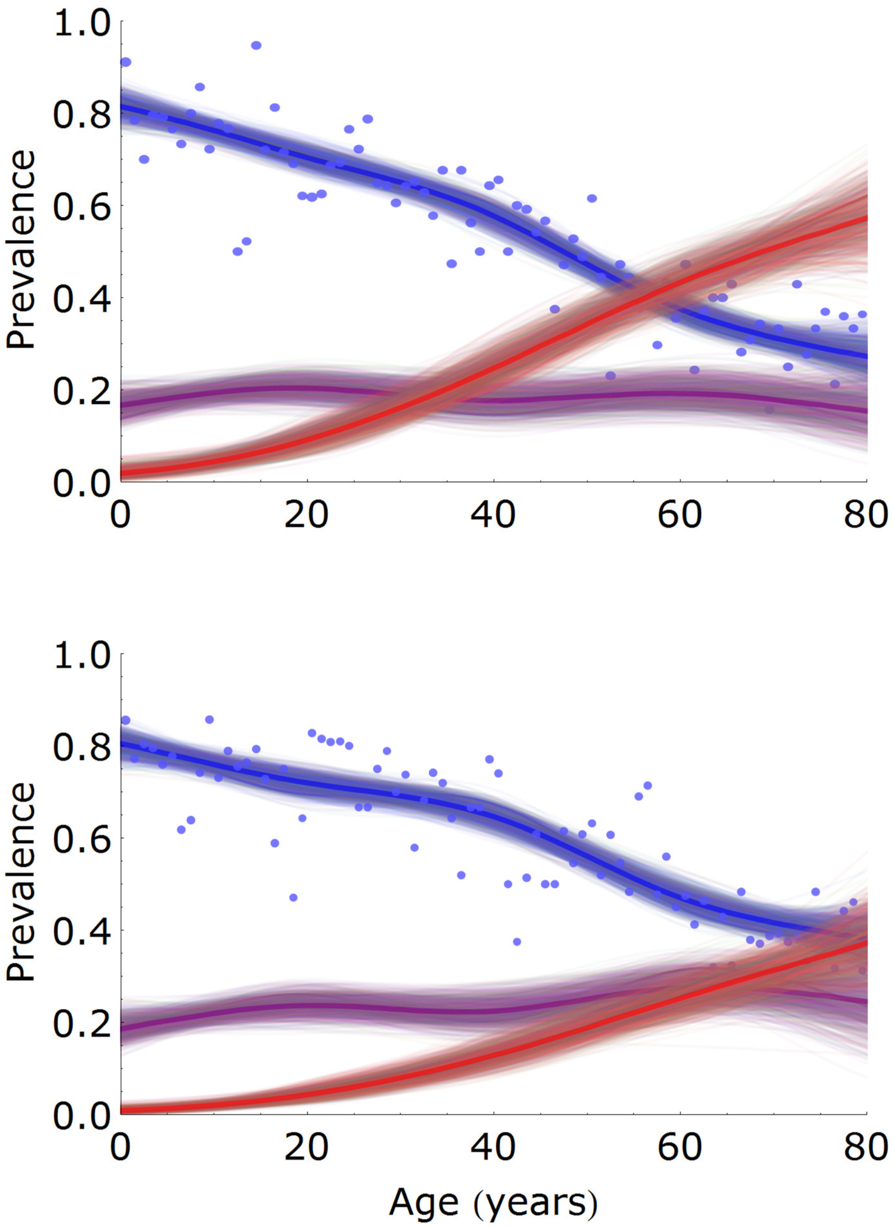

Fig 4. Estimation of age- and sex-specific prevalence. Prevalence estimates are presented for females (top panel) and males (bottom panel), and for classes of low (susceptible, blue), intermediate (latently infected, purple), and high (latently infected with increased antibodies, red) antibody measurements. Shown are 1,000 samples from the posterior distribution (thin lines) with posterior medians (bold lines). Dots indicate the fraction of samples that would be classified as uninfected with the cut-off specified by the supplier of the assay. The number of samples per 1-year age group is approximately 35 (females) and 30 (males).

https://doi.org/10.1371/journal.pcbi.1005719.g004

prevalence of persons with increased antibodies increases from 0.09 (95%CrI: 0.06-0.13) at 20 years to 0.57 (95%CrI: 0.47-0.67) at 80 years in females, and from 0.04 (95%CrI: 0.03-0.07) to 0.37 (95%CrI: 0.28-0.46) in males. Hence, in older persons the prevalence of persons with increased antibodies is 54% (or 20 per cent points) higher in females than in males.

Of particular interest is the prevalence of infection in females of childbearing age, as this group is at risk of transmission to the fetus or newborn. Using the above analyses, we find that

the prevalence of infection (i.e. the combined prevalence in the L and B compartments) is 0.30 (95%CrI: 0.27-0.33) in 20-year-old females and 0.42 (95%CrI: 0.39-0.46) in 40-year-old females. If we combine these figures with the observation that approximately 20% of children are infected at six months of age, and that less than 5% of children in the Netherlands in 2007 had a mother under 20 years or over 40 years, we deduce that the probability of perinatal transmission could be between 0.20/0.42 = 0.48 and 0.20/0.30 = 0.67, with the exact figure depending on the distribution of ages at which mothers give birth. In addition, one could envisage that the highest risk of (severe) infection of the fetus or newborn is when mothers are infected or experience a reactivation episode. The estimated rates at which susceptible females of 20 and 40 years are infected are 0.0055 per year (95%CrI: 0.0036-0.0077) and 0.0092 per year (95%CrI: 0.0069-0.011) per year, respectively. The rates at which latently infected females of 20 and 40 years are re-infected or experience a reactivation episode are of similar magnitude, and are estimated at 0.0059 per year (95%CrI: 0.0038-0.0086) and 0.0093 per year (95%CrI: 0.0064-0.012), respectively. The overall rates of infection, reactivation, and re-infection in 20 and 40 year-old females are given by the sum of the above estimates, and are approximately 1% and 2% per year, respectively.

Estimation of reactivation and re-infection rates

To evaluate the ability of different transmission hypotheses explaining the data, and to obtain parameter estimates that have a biological interpretation, we analyzed the data with transmission models. A comparison of model scenarios based on the information criteria WAIC and WBIC is given in Table 1. Overall, the analyses show that models with the possibility of multiple infectious reactivations perform best (Models E and F; lowest WAIC and WBIC), that models with at most one infectious reactivation perform worse (Models A and B; ΔWAIC and ΔWBIC $\approx 10-15$), and that models without reactivation or with reactivation not being infectious have very low support (Models C, D, and G). These results indicate that infectious reactivation is key to adequately explain the data with transmission models. This is true in our model with contact structure based on reported physical contacts [32], and also in an alternative model formulation that assumes a uniform contact structure (ΔWAIC = 151.9 in favor of the model with reactivation over the model without reactivation and no re-infection).

Within the set of models with infectious reactivation there are only small differences between models that do and do not incorporate re-infection (Model A versus Model B, and Model E versus Model F). This indicates that while infectious reactivation is essential to

Table 1. Model selection of transmission scenarios.

Model	Description	WAIC	ΔWAIC	WBIC	ΔWBIC
A	Reactivation and re-infection	22156.2	13.0	22211.3	13.7
B	No re-infection	22155.5	12.3	22209.4	11.8
C	No reactivation	22363.6	220.4	22396.9	199.3
D	Reactivation/re-infection not infectious	22215.3	72.1	22247.9	36.6
E	Multiple reactivations/re-infections	22144.0	0.7	22197.6	0
F	Multiple reactivations and no re-infection	22143.2	0	22198.4	0.8
G	Multiple re-infections and no reactivation	22364.4	221.2	22396.8	199.2

For each of seven model scenarios we report the WAIC, a measure of predictive performance, and WBIC, a measure for the most likely model generating the data. Also shown are the WAIC and WBIC differences with the best fitting model. Models E-G contain the possibility of multiple reactivation/re-infection events in persons with increased antibody concentrations (the B compartment; cf. Fig 2).

https://doi.org/10.1371/journal.pcbi.1005719.t001

adequately describe the data, the analyses are inconclusive with respect to whether or not infectious re-infection should be included.

Fig 5 and Table 2 show parameter estimates of the model with highest statistical support (as judged by WBIC). The preferred model (Model E) includes multiple reactivations and re-infections, infectious reactivation, and infectious re-infection. In this model, the estimated transmissibility of primary infection (β_1) is much lower than the transmissibility of reactivation/re-infection (β_2). In fact, the posterior median of β_2 is more than an order of magnitude larger than the posterior median of β_1. Further, the relative susceptibility to re-infection (i.e. the probability of re-infection in a contact that would lead to infection if the contacted person were uninfected) has a broad posterior distribution, and cannot be estimated with meaningful precision from the data ($\hat{z} = 0.32$; 95%CrI: 0.017-0.84). Similar findings are obtained in other model scenarios, in particular Models A-B and E-F (Table 1).

Estimates of the reactivation rates are quantitatively close in models with high support (Models E-F). Reactivation rates generally increase with increasing age, and are substantially

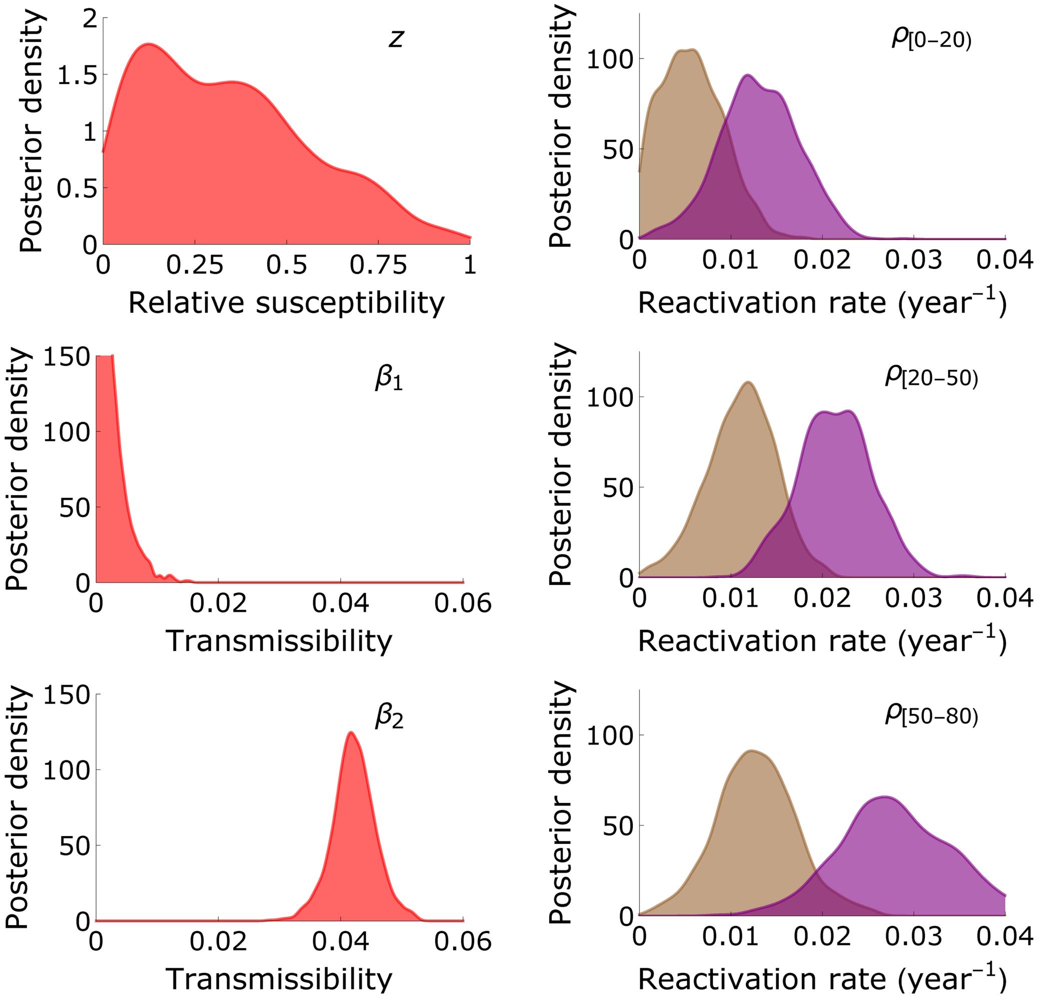

Fig 5. Parameter estimates. Shown are kernel-smoothed posterior distributions of the relative susceptibility to re-infection (z), the transmissibility of primary infection (β_1) and reactivation/re-infection (β_2), and the reactivation rates in persons aged 0-20 years ($\rho_{[0,20)}$, purple: females; brown: males), 20-50 years ($\rho_{[20,50)}$), and 50-80 years ($\rho_{[50-80)}$) of the transmission model with the possibility of multiple reactivations and re-infections (Model E in Table 1).

https://doi.org/10.1371/journal.pcbi.1005719.g005

Table 2. Parameter estimates of the transmission model.

Parameter	Description	Median	95%CrI
β_1	Transmissibility of primary infection	0.0019	0.000081—0.0089
β_2	Transmissibility of re-infection/reactivation	0.042	0.035—0.049
z	Relative susceptibility for re-infection	0.32	0.017—0.84
$\rho^{♀}_{[0,20)}$	Reactivation rate in 0-20 year old females (yr^{-1})	0.013	0.0042—0.021
$\rho^{♀}_{[20,50)}$	Reactivation rate in 20-50 year old females (yr^{-1})	0.021	0.013—0.029
$\rho^{♀}_{[50,80)}$	Reactivation rate in 50-80 year old females (yr^{-1})	0.028	0.017—0.040
$\rho^{♂}_{[0,20)}$	Reactivation rate in 0-20 year old males (yr^{-1})	0.0054	0.0035—0.013
$\rho^{♂}_{[20,50)}$	Reactivation rate in 20-50 year old males (yr^{-1})	0.011	0.0035—0.018
$\rho^{♂}_{[50,80)}$	Reactivation rate in 50-80 year old males (yr^{-1})	0.013	0.0043—0.021
μ_ρ	Hyperparameter for the reactivation rate (mean)	0.015	0.0047—0.027
σ_ρ	Hyperparameter for the reactivation rate (sd)	0.010	0.0050—0.028

Parameters estimates (represented by posterior medians with 95% credible intervals) are shown of the transmission model with reactivation and re-infection in the B compartment (model E). This model gives the best fit to the data, as judged by WBIC and WAIC (Table 1). For all parameters the potential scale reduction factor Q is close to 1 ($0.999 < Q < 1.001$), and effective sample sizes of the parameters n_{eff} range from 3596 (β_1) to 4457 (μ_ρ).

https://doi.org/10.1371/journal.pcbi.1005719.t002

higher in females than in males. In the preferred model (Model E), the estimated reactivation rate is 0.013 per year (95%CrI: 0.0042-0.021) in 0-20 year-old females, which increases to 0.021 per year (95%CrI: 0.013-0.029) in 20-50 year-old females, and then increases further to 0.028 per year (95%CrI: 0.017-0.040) in 50 + -year-old females (Table 2). The corresponding reactivation rates in males are 0.0054 per year (95%CrI: 0.0035-0.013), 0.011 per year (95%CrI: 0.0035-0.018), and 0.013 per year (95%CrI: 0.0043-0.021). These estimates are slightly higher and slightly more precise in the model without re-infection (Model F), and somewhat higher in models with a single reactivation/re-infection event (Models A-B).

In the two models with highest support (Models E-F), estimates of the force of infection increase from approximately 0.012-0.013 per year in the youngest age group to 0.014-0.017 per year in 10-15 year-old girls (Fig 6). Owing to the slightly higher contact rates in females than in men, the estimated force of infection is usually slightly higher in females than in males in the age groups 10-25 years [32]. In older age groups, estimates of the forces of infection decrease to lower values (≈0.01 per year). Noteworthy, the extreme age-specific differences in the force of infection usually observed for directly transmitted infectious diseases, with high infection rates in children and much lower rates in adults, are much less pronounced here due to infectious reactivation in older age strata combined with age-assortative mixing [32, 34, 35].

In models with re-infection, estimates of re-infection rate ($z\lambda^i(a)$) are considerably smaller than estimates of the reactivation rates ($\rho^i(a)$) because the estimated forces of infection ($\lambda^i(a)$) are usually lower than the reactivation rates, especially in females (Fig 6). Hence, re-infection contributes little to boosting of the antibody concentrations in those age groups where most of the boosting occurs (>20 years; Fig 4). In fact, in adult females it is not uncommon that the reactivation rate is more than an order of magnitude higher than the estimated re-infection rate ($\log_{10}(\rho^{♀}(a)/(z\lambda^{♀}(a))) > 1$).

Discussion

Our study of population-wide serological data shows that IgG antibody concentrations contain a wealth of information on the transmission dynamics of CMV. Specifically, the analyses reveal that (i) the prevalence of CMV increases gradually with age such that at old age the majority of

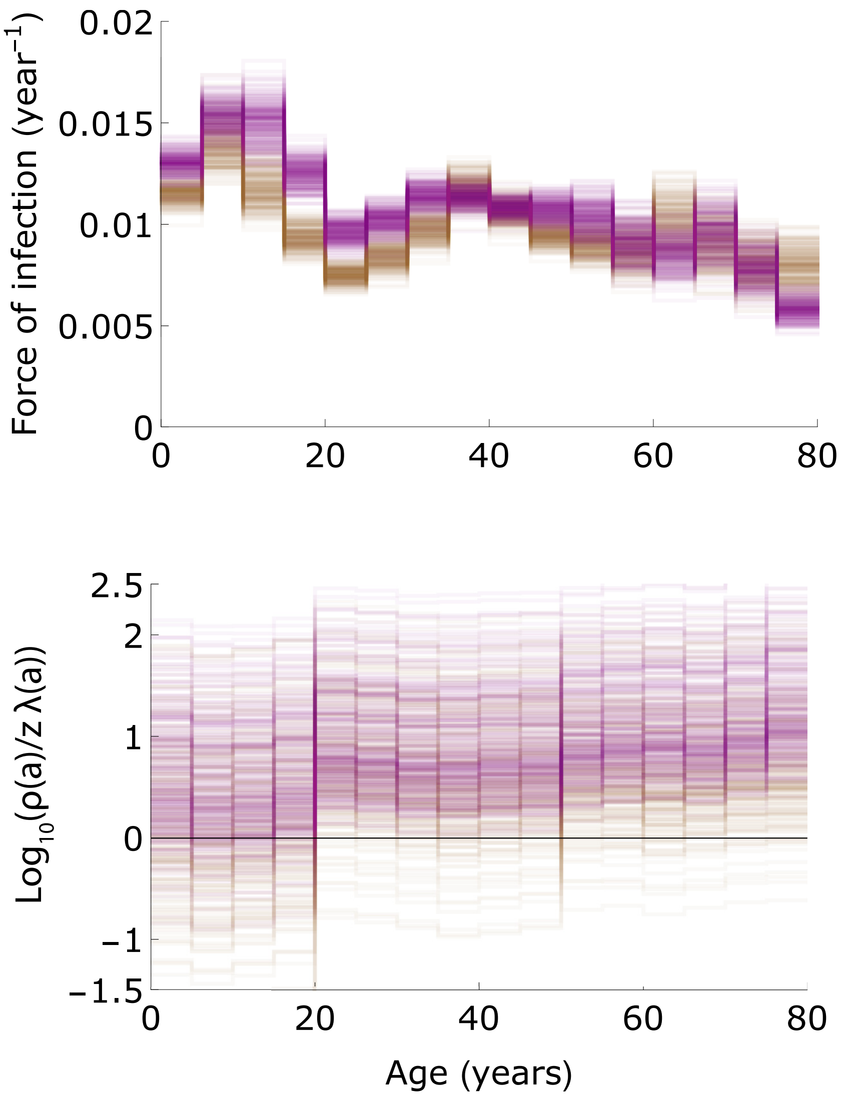

Fig 6. The force of infection and magnitude of reactivation relative to re-infection. The top panel shows posterior estimates of the forces of infection in females and males in Model E (Table 1; purple: females; brown: males). The bottom panel shows the $\log_{10}$ of the reactivation rates divided by the re-infection rates ($\rho^i(a)/z\lambda^i(a)$ with $i \in \{♀, ♂\}$). Results are shown for 250 samples from the posterior distribution.

https://doi.org/10.1371/journal.pcbi.1005719.g006

persons in the Netherlands are infected; (ii) except for the very young, the prevalence of CMV is systematically higher in females than in males. This is mainly due to a higher incidence of infection in adult women than in adult men of similar age; (iii) antibody concentrations in seropositive (i.e. infected) persons increase monotonically with age, especially in women; (iv) the above findings (i)-(iii) cannot be explained by simple transmission models in which only primary infection is infectious. This is caused by the fact that transmissibility of primary infection determines the rate at which age-specific prevalence increases; if transmissibility of primary infection would be high then a high prevalence of infection is expected in children. In other words, the fact that seroprevalence increases gradually with age puts an upper bound on the force of infection, and this in turn constrains the transmissibility of primary infection to low values.

While aforementioned findings (i)-(iii) have been noticed before in other settings ([1] and references therein, [21]), our analyses are the first to provide precise estimates using a large

population sample. Moreover, the results lead us to a new transmission hypothesis in which infectious reactivation is a key driver of transmission of CMV in the population. Since several other studies have found a gradual increase in seroprevalence [1], this explanation may not be restricted to the Dutch situation, but hold in general. Underpinning this hypothesis, next to the well-known observations of shedding of CMV in breast milk and cervical material in the third trimester of pregnancy [45–47], detectable virus also has been found in healthy adults in one study [24], while in another study CMV DNA has been detected in urine of the majority of older persons [23].

The main implication is that the majority of CMV infections may not be caused by transmission among children after primary infection, even though levels of shedding can be high in infants [46, 48], but rather by older persons who go through one or more reactivation episodes. This contrasts with common childhood diseases such as measles, mumps, rubella, and pertussis. For these pathogens, infection in unvaccinated populations generally occurs at a young age, and children are the drivers of transmission. It also contrasts with other herpes viruses such as varicella zoster virus and Epstein-Bar virus for which well over 50% of the population is infected at the age of 10 years [34]. It may be comparable with other herpes viruses such as HSV1 and HSV2, which show a slowly increasing age-specific seroprevalence [49]. A corollary is that persistence of CMV in the population is not possible with transmission from primary infected persons only, and is dependent on infectious reactivation. Currently, we are focusing on making this idea more precise by calculation of the basic reproduction number, and the reproduction numbers of perinatal transmission, primary infection, and reactivation [50]. This will help put bounds on the relative contribution of each of the transmission routes.

With infectious reactivation and perinatal infection being putative drivers of transmission, it is to be expected that elimination by vaccination may prove more difficult than for directly transmitted pathogens, as it will require the pool of latently infected persons to dwindle to zero by demographic turnover. This can take up to the lifetime of one generation, and perhaps more if vaccination cannot prevent perinatal transmission to infants who are too young for vaccination. Thus, a question is whether vaccination formulations and strategies exist that minimize the probability of transmission to young infants. This is all the more of importance as a main source of morbidity is by congenital infection, and the timescale on which reductions in congenital disease are expected determines the projected health impact of vaccination [51]. In this context, next to the ability of a vaccine to prevent infection it may perhaps be equally important that a vaccine is able to reduce the probability of reactivation. Such reductions are likely mediated by T-cell responses of the host, and several (but not all) vaccines under development are expected to induce boosting of T-cell immune responses [52–54].

A number of limitations and assumptions deserve scrutiny. First, the transmission model analyses assume that the population is in endemic equilibrium. For a single cross-sectional data set such as the one considered in the present study this assumption is unavoidable if one does not want to introduce additional parameters that cannot be estimated by the data. Reassuringly, the patterns of infection present in the serological data have been found in several serological studies carried out in high-income countries over the past decades [1]. Also, no systematic patterns of increasing or decreasing seroprevalence over time have been found, and this is further reason to believe that there have not been major changes in the epidemiology of CMV over time [1]. Second, we assume that antibody measurements not only give information on CMV infection status, but also whether or not reactivation or re-infection have taken place. Unfortunately, there is no direct empirical evidence confirming or falsifying this assumption, and this is an area where in-depth comparison of the infection and immune status of persons with low and high antibody concentrations is urgently needed. Third, the analyses assume that person-to-person transmission is proportional to observed human contact patterns [32, 33].

Although this assumption is commonly made and has met with considerable success (e.g., [33, 44, 55, 56]), it is conceivable that transmission of CMV does not abide by the social contact hypothesis, and that a more complex contact structure would be able to explain the patterns of seroprevalence in a simple transmission model. To investigate the impact of the contact structure, we have analyzed transmission models with a uniform contact structure, and found that models with infectious reactivation still provide the best fit to the data (ΔWAIC > 100; Results). As a final limitation we would like to add that, in principle, it is conceivable that the data can be explained alternatively by an intricate interplay between variation in the susceptibility to infection in conjunction with age-specific variations in the strength of the antibody response. Alas, evidence for or against this hypothesis is lacking.

Our inferential analyses indicate that the transmissibility of primary infection is much lower than the transmissibility after reactivation. This seems to be at odds with the observation that prolonged and high-level virus shedding can occur in bodily fluids after primary infection in children [46, 47]. However, it could be that transitions from the infected class to the infected class with increased antibodies are in effect not the result of a single reactivation or re-infection event, but rather the result of multiple underlying reactivations or re-infections. If this were true, as seems plausible, estimates of the reactivation and re-infection rates as well as the transmissibility of reactivation and re-infection should be interpreted as compound parameters that take into account multiple reactivations and re-infections occurring over the lifetime of an infected person.

Acknowledgments

We thank Sophia de Jong (VU University Amsterdam) and Can Keşmir (Utrecht University) for discussion and critical reading, and the persons included in the PIENTER2 study for their participation.

Author Contributions

Conceptualization: Michiel van Boven, Ann C. Vossen, Debbie van Baarle.

Data curation: Marjolein J. Korndewal.

Formal analysis: Michiel van Boven, Jan van de Kassteele, Christiaan H. van Dorp.

Funding acquisition: Fiona van der Klis, Hester E. de Melker.

Investigation: Michiel van Boven.

Methodology: Michiel van Boven, Jan van de Kassteele, Christiaan H. van Dorp, Mirjam Kretzschmar.

Resources: Marjolein J. Korndewal.

Software: Michiel van Boven, Jan van de Kassteele, Christiaan H. van Dorp.

Validation: Michiel van Boven.

Visualization: Michiel van Boven.

Writing – original draft: Michiel van Boven.

Writing – review & editing: Michiel van Boven, Jan van de Kassteele, Marjolein J. Korndewal, Christiaan H. van Dorp, Mirjam Kretzschmar, Fiona van der Klis, Hester E. de Melker, Ann C. Vossen, Debbie van Baarle.

References

1. Cannon MJ, Schmid DS, Hyde TB. Review of cytomegalovirus seroprevalence and demographic characteristics associated with infection. Rev Med Virol. 2010; 20(4):202–213. https://doi.org/10.1002/rmv.655 PMID: 20564615
2. Dollard SC, Grosse SD, Ross DS. New estimates of the prevalence of neurological and sensory sequelae and mortality associated with congenital cytomegalovirus infection. Rev Med Virol. 2007; 17(5):355–363. https://doi.org/10.1002/rmv.544 PMID: 17542052
3. Kenneson A, Cannon MJ. Review and meta-analysis of the epidemiology of congenital cytomegalovirus (CMV) infection. Rev Med Virol. 2007; 17(4):253–276. https://doi.org/10.1002/rmv.535 PMID: 17579921
4. Griffiths P, Plotkin S, Mocarski E, Pass R, Schleiss M, Krause P, et al. Desirability and feasibility of a vaccine against cytomegalovirus. Vaccine. 2013; 31 Suppl 2:197–203. https://doi.org/10.1016/j.vaccine.2012.10.074
5. Ramanan P, Razonable RR. Cytomegalovirus infections in solid organ transplantation: a review. Infect Chemother. 2013; 45(3):260–271. https://doi.org/10.3947/ic.2013.45.3.260 PMID: 24396627
6. Roberts ET, Haan MN, Dowd JB, Aiello AE. Cytomegalovirus antibody levels, inflammation, and mortality among elderly Latinos over 9 years of follow-up. Am J Epidemiol. 2010; 172(4):363–371. https://doi.org/10.1093/aje/kwq177 PMID: 20660122
7. Gkrania-Klotsas E, Langenberg C, Sharp SJ, Luben R, Khaw KT, Wareham NJ. Higher immunoglobulin G antibody levels against cytomegalovirus are associated with incident ischemic heart disease in the population-based EPIC-Norfolk cohort. J Infect Dis. 2012; 206(12):1897–1903. https://doi.org/10.1093/infdis/jis620 PMID: 23045624
8. Boeckh M, Geballe AP. Cytomegalovirus: pathogen, paradigm, and puzzle. J Clin Invest. 2011; 121(5):1673–1680. https://doi.org/10.1172/JCI45449 PMID: 21659716
9. Pawelec G, Derhovanessian E. Role of CMV in immune senescence. Virus Res. 2011; 157(2):175–179. https://doi.org/10.1016/j.virusres.2010.09.010 PMID: 20869407
10. Pawelec G. Immunosenenescence: role of cytomegalovirus. Exp Gerontol. 2014; 54:1–5. https://doi.org/10.1016/j.exger.2013.11.010 PMID: 24291068
11. Sansoni P, Vescovini R, Fagnoni FF, Akbar A, Arens R, Chiu YL, et al. New advances in CMV and immunosenescence. Exp Gerontol. 2014; 55:54–62. https://doi.org/10.1016/j.exger.2014.03.020 PMID: 24703889
12. Klenerman P, Oxenius A. T cell responses to cytomegalovirus. Nat Rev Immunol. 2016; 16(6):367–377. https://doi.org/10.1038/nri.2016.38 PMID: 27108521
13. Derhovanessian E, Maier AB, Hahnel K, McElhaney JE, Slagboom EP, Pawelec G. Latent infection with cytomegalovirus is associated with poor memory CD4 responses to influenza A core proteins in the elderly. J Immunol. 2014; 193(7):3624–3631. https://doi.org/10.4049/jimmunol.1303361 PMID: 25187662
14. Frasca D, Diaz A, Romero M, Landin AM, Blomberg BB. Cytomegalovirus (CMV) seropositivity decreases B cell responses to the influenza vaccine. Vaccine. 2015; 33(12):1433–1439. https://doi.org/10.1016/j.vaccine.2015.01.071 PMID: 25659271
15. Frasca D, Blomberg BB. Aging, cytomegalovirus (CMV) and influenza vaccine responses. Hum Vaccin Immunother. 2016; 12(3):682–690. https://doi.org/10.1080/21645515.2015.1105413 PMID: 26588038
16. Sung H, Schleiss MR. Update on the current status of cytomegalovirus vaccines. Expert Rev Vaccines. 2010; 9(11):1303–1314. https://doi.org/10.1586/erv.10.125 PMID: 21087108
17. Plotkin S. The history of vaccination against cytomegalovirus. Med Microbiol Immunol. 2015; 204(3):247–254. https://doi.org/10.1007/s00430-015-0388-z PMID: 25791890
18. Staras SA, Dollard SC, Radford KW, Flanders WD, Pass RF, Cannon MJ. Seroprevalence of cytomegalovirus infection in the United States, 1988-1994. Clin Infect Dis. 2006; 43(9):1143–1151. https://doi.org/10.1086/508173 PMID: 17029132
19. Staras SA, Flanders WD, Dollard SC, Pass RF, McGowan JE, Cannon MJ. Cytomegalovirus seroprevalence and childhood sources of infection: A population-based study among pre-adolescents in the United States. J Clin Virol. 2008; 43(3):266–271. https://doi.org/10.1016/j.jcv.2008.07.012 PMID: 18778968
20. Bate SL, Dollard SC, Cannon MJ. Cytomegalovirus seroprevalence in the United States: the national health and nutrition examination surveys, 1988-2004. Clin Infect Dis. 2010; 50(11):1439–1447. https://doi.org/10.1086/652438 PMID: 20426575
21. Korndewal MJ, Mollema L, Tcherniaeva I, van der Klis F, Kroes AC, Oudesluys-Murphy AM, et al. Cytomegalovirus infection in the Netherlands: seroprevalence, risk factors, and implications. J Clin Virol. 2015; 63:53–58. https://doi.org/10.1016/j.jcv.2014.11.033 PMID: 25600606

22. Aberle JH, Puchhammer-Stockl E. Age-dependent increase of memory B cell response to cytomegalovirus in healthy adults. Exp Gerontol. 2012; 47(8):654–657. https://doi.org/10.1016/j.exger.2012.04.008 PMID: 22564865

23. Stowe RP, Kozlova EV, Yetman DL, Walling DM, Goodwin JS, Glaser R. Chronic herpesvirus reactivation occurs in aging. Exp Gerontol. 2007; 42(6):563–570. https://doi.org/10.1016/j.exger.2007.01.005 PMID: 17337145

24. Parry HM, Zuo J, Frumento G, Mirajkar N, Inman C, Edwards E, et al. Cytomegalovirus viral load within blood increases markedly in healthy people over the age of 70 years. Immun Ageing. 2016; 13:1. https://doi.org/10.1186/s12979-015-0056-6 PMID: 26734066

25. Alonso Arias R, Moro-Garcia MA, Echeverria A, Solano-Jaurrieta JJ, Suarez-Garcia FM, Lopez-Larrea C. Intensity of the humoral response to cytomegalovirus is associated with the phenotypic and functional status of the immune system. J Virol. 2013; 87(8):4486–4495. https://doi.org/10.1128/JVI.02425-12 PMID: 23388717

26. de Bourcy CF, Angel CJ, Vollmers C, Dekker CL, Davis MM, Quake SR. Phylogenetic analysis of the human antibody repertoire reveals quantitative signatures of immune senescence and aging. Proc Natl Acad Sci USA. 2017; 114(5):1105–1110. https://doi.org/10.1073/pnas.1617959114 PMID: 28096374

27. van der Klis FR, Mollema L, Berbers GA, de Melker HE, Coutinho RA. Second national serum bank for population-based seroprevalence studies in the Netherlands. Netherlands Journal of Medicine. 2009; 67:301–8. PMID: 19687529

28. Plummer M. JAGS: A program for analysis of Bayesian graphical models using Gibbs sampling; 2003.

29. R Core Team. R: A Language and Environment for Statistical Computing; 2016. Available from: https://www.R-project.org/.

30. Diekmann O, Heesterbeek H, Britton T. Mathematical Tools for Understanding Infectious Disease Dynamics. Princeton University Press; 2013.

31. Farrington CP, Whitaker HJ. Estimation of effective reproduction numbers for infectious diseases using serological survey data. Biostatistics. 2003; 4(4):621–632. https://doi.org/10.1093/biostatistics/4.4.621 PMID: 14557115

32. van de Kassteele J, van Eijkeren J, Wallinga J. Efficient estimation of age-specific social contact rates between men and women. Annals of Applied Statistics. 2017; 11:320–339. https://doi.org/10.1214/16-AOAS1006

33. Mossong J, Hens N, Jit M, Beutels P, Auranen K, Mikolajczyk R, et al. Social contacts and mixing patterns relevant to the spread of infectious diseases. PLOS Medicine. 2008; 5:e74. https://doi.org/10.1371/journal.pmed.0050074 PMID: 18366252

34. van Lier A, Lugner A, Opstelten W, Jochemsen P, Wallinga J, Schellevis F, et al. Distribution of Health Effects and Cost-effectiveness of Varicella Vaccination are Shaped by the Impact on Herpes Zoster. EBioMedicine. 2015; 2(10):1494–1499. https://doi.org/10.1016/j.ebiom.2015.08.017 PMID: 26629544

35. Hens N, Shkedy Z, Aerts M, Faes C, Van Damme P, Beutels P. Modeling Infectious Disease Parameters Based on Serological and Social Contact Data. Springer New York; 2012.

36. Goeyvaerts N, Hens N, Ogunjimi B, Aerts M, Shkedy Z, van Damme P, et al. Estimating infectious disease parameters from data on social contacts and serological status. Journal of the Royal Statistical Society: Series C (Applied Statistics). 2010; 59(2):255–277. https://doi.org/10.1111/j.1467-9876.2009.00693.x

37. Goeyvaerts N, Hens N, Aerts M, Beutels P. Model structure analysis to estimate basic immunological processes and maternal risk for parvovirus B19. Biostatistics (Oxford, England). 2011; 12(2):283–302. https://doi.org/10.1093/biostatistics/kxq059

38. Gelman A, Rubin DB. Inference from iterative simulation using multiple sequences. Statistical Science. 1992; 7(4):457–472. https://doi.org/10.1214/ss/1177011136

39. Watanabe S. A widely applicable Bayesian information criterion. Journal of Machine Learning Research. 2013; 14:867–897.

40. Piironen J, Vehtari A. Comparison of Bayesian predictive methods for model selection. Statistics and Computing. 2016; p. 1–25.

41. Vehtari A, Gelman A, Gabry J. Practical Bayesian model evaluation using leave-one-out cross-validation and WAIC. Statistics and Computing. 2016; in press.

42. Steens A, Waaijenborg S, Teunis PF, Reimerink JH, Meijer A, van der Lubben M, et al. Age-dependent patterns of infection and severity explaining the low impact of 2009 influenza A (H1N1): evidence from serial serologic surveys in the Netherlands. Am J Epidemiol. 2011; 174(11):1307–1315. https://doi.org/10.1093/aje/kwr245 PMID: 22025354

43. te Beest D, de Bruin E, Imholz S, Wallinga J, Teunis P, Koopmans M, et al. Discrimination of influenza infection (A/2009 H1N1) from prior exposure by antibody protein microarray analysis. PLoS ONE. 2014; 9(11):e113021. https://doi.org/10.1371/journal.pone.0113021 PMID: 25405997

44. te Beest DE, Birrell PJ, Wallinga J, De Angelis D, van Boven M. Joint modelling of serological and hospitalization data reveals that high levels of pre-existing immunity and school holidays shaped the influenza A pandemic of 2009 in the Netherlands. J R Soc Interface. 2015; 12(103). https://doi.org/10.1098/rsif.2014.1244 PMID: 25540241

45. Hamprecht K, Maschmann J, Vochem M, Dietz K, Speer CP, Jahn G. Epidemiology of transmission of cytomegalovirus from mother to preterm infant by breastfeeding. Lancet. 2001; 357(9255):513–518. https://doi.org/10.1016/S0140-6736(00)04043-5 PMID: 11229670

46. Cannon MJ, Hyde TB, Schmid DS. Review of cytomegalovirus shedding in bodily fluids and relevance to congenital cytomegalovirus infection. Rev Med Virol. 2011; 21(4):240–255. https://doi.org/10.1002/rmv.695 PMID: 21674676

47. Pass RF, Anderson B. Mother-to-Child Transmission of Cytomegalovirus and Prevention of Congenital Infection. J Pediatric Infect Dis Soc. 2014; 3 Suppl 1:2–6. https://doi.org/10.1093/jpids/piu069

48. Cannon MJ, Stowell JD, Clark R, Dollard PR, Johnson D, Mask K, et al. Repeated measures study of weekly and daily cytomegalovirus shedding patterns in saliva and urine of healthy cytomegalovirus-seropositive children. BMC Infect Dis. 2014; 14:569. https://doi.org/10.1186/s12879-014-0569-1 PMID: 25391640

49. Woestenberg PJ, Tjhie JH, de Melker HE, van der Klis FR, van Bergen JE, van der Sande MA, et al. Herpes simplex virus type 1 and type 2 in the Netherlands: seroprevalence, risk factors and changes during a 12-year period. BMC Infect Dis. 2016; 16:364. https://doi.org/10.1186/s12879-016-1707-8 PMID: 27484304

50. de Jong S. Estimation of Perinatal Transmission Rates of Cytomegalovirus From Serological Data and Calculation of Reproduction Numbers. MSc thesis, VU University Amsterdam; 2017.

51. Korndewal MJ, Vossen AC, Cremer J, VAN Binnendijk RS, Kroes AC, van der Sande MA, et al. Disease burden of congenital cytomegalovirus infection at school entry age: study design, participation rate and birth prevalence. Epidemiol Infect. 2016; 144(7):1520–1527. https://doi.org/10.1017/S0950268815002708 PMID: 26554756

52. Sabbaj S, Pass RF, Goepfert PA, Pichon S. Glycoprotein B vaccine is capable of boosting both antibody and CD4 T-cell responses to cytomegalovirus in chronically infected women. J Infect Dis. 2011; 203(11):1534–1541. https://doi.org/10.1093/infdis/jir138 PMID: 21592981

53. Bialas KM, Permar SR. The March towards a Vaccine for Congenital CMV: Rationale and Models. PLoS Pathog. 2016; 12(2):e1005355. https://doi.org/10.1371/journal.ppat.1005355 PMID: 26866914

54. Schleiss MR. Preventing Congenital Cytomegalovirus Infection: Protection to a'T'. Trends Microbiol. 2016; 24(3):170–172. https://doi.org/10.1016/j.tim.2016.01.007 PMID: 26857178

55. Wallinga J, Teunis P, Kretzschmar M. Using data on social contacts to estimate age-specific transmission parameters for respiratory-spread infectious agents. Am J Epidemiol. 2006; 164(10):936–944. https://doi.org/10.1093/aje/kwj317 PMID: 16968863

56. Goeyvaerts N, Willem L, Van Kerckhove K, Vandendijck Y, Hanquet G, Beutels P, et al. Estimating dynamic transmission model parameters for seasonal influenza by fitting to age and season-specific influenza-like illness incidence. Epidemics. 2015; 13:1–9. https://doi.org/10.1016/j.epidem.2015.04.002 PMID: 26616037
